# Work-related stress among emergency medical services healthcare workers in İstanbul: A cross-sectional analysis

**DOI:** 10.1097/MD.0000000000047751

**Published:** 2026-02-28

**Authors:** Derya Abuşka, Yilmaz Aydin, Hasan Yasin Soylu, Verda Tunaligil, Doğaç Niyazi Özüçelik

**Affiliations:** a Department of Emergency Medicine, Republic of Türkiye Ministry of Health (TR MoH) University of Health Sciences, İstanbul Training and Research Hospital, İstanbul, Türkiye; b Presidency of Disaster Health and Emergency Medical Services, TR MoH Health Directorate of İstanbul, İstanbul, Türkiye; c Department of Emergency Medicine, TR MoH University of Health Sciences, Başakşehir Çam Sakura City Hospital, İstanbul, Türkiye; d Disaster Health and Emergency Medical Services, TR MoH Health Directorate of İstanbul, İstanbul, Türkiye; e Department of Emergency Medicine, Faculty of Medicine, Haliç University, İstanbul, Türkiye.

**Keywords:** emergency medical services, healthcare workers, İstanbul, organizational stress, work-related stress

## Abstract

Emergency medical services healthcare workers (EMS-HCWs) are frequently exposed to high occupational stress due to demanding work environments, which may adversely affect their psychological well-being and job performance. Limited data are available for Türkiye, particularly for İstanbul, which is a densely populated metropolitan city with a large EMS workforce. This cross-sectional analytical study was conducted between September and December of 2014 in İstanbul. Data were collected using the Perceived Stress Scale (PSS-14) and Organizational Stress Questionnaire-Doetinchem (VOS-D). Of the 1100 EMS-HCWs invited to participate, 1000 returned the completed questionnaires (response rate: 90.9%). After excluding incomplete forms, 574 responses were included in the final analysis (inclusion rate: 52.2%). Descriptive statistics, nonparametric group comparisons, effect size estimation, correlation analyses, and multiple linear regression were performed. Overall, 78.6% of EMS-HCWs demonstrated moderate levels of perceived stress. Female EMS-HCWs reported significantly higher Perceived Stress Scale and Organizational Stress Questionnaire-Doetinchem scores compared with males (all *P* < .001). Command and Control Center (CCC) staff had higher scores for psychological complaints, health problems, and social variables than the station staff (all *P* < .05). Workplace and behavioral factors, including inadequate training, limited social support, perceived staffing shortages, low job satisfaction, unsafe working conditions, smoking, and alcohol use, were significantly associated with higher stress scores. Regression analysis revealed that the VOS-D subdomains (stressors, psychological complaints, and social variables) significantly predicted the Perceived Stress Scale total scores, explaining 23.4% of the variance (*P* < .001). Emergency medical services healthcare workers in İstanbul experienced substantial occupational stress, with female EMS-HCWs and Command and Control Center staff being particularly vulnerable. These findings highlight the urgent need for targeted and multifaceted interventions to improve workplace safety, staffing adequacy, and psychosocial support, thereby promoting the health and effectiveness of EMS-HCWs in high-density urban settings.

## 1. Introduction

### 1.1. Background

Emergency medical service healthcare workers (EMS-HCWs) play a pivotal role in society by providing essential care during emergencies. However, the demanding nature of their work often leads to high stress levels that adversely affect both physical and psychological health. Constant exposure to traumatic events, long working hours, and life-or-death situations have been shown to significantly impact mental well-being.^[1,2]^ Addressing the work-related stress of this group is therefore essential to safeguard their health and ensure sustainable, high-quality emergency care provision.^[3,4]^

A meta-analysis of 27 international studies involving 30,878 EMS-HCWs reported the prevalence of post-traumatic stress, depression, anxiety, and psychological distress as 11%, 15%, 15%, and 27%, respectively.^[5]^ A systematic review further indicated that dysfunctional coping styles, certain personality traits, and frequent exposure to critical incidents contribute to stress development.^[6]^ While these findings highlight a global burden, stress levels vary across cultural and organizational contexts, emphasizing the importance of localized research.

Despite growing international evidence, studies from Türkiye remain scarce, particularly in İstanbul – a densely populated metropolitan area with a disproportionately high EMS workload. To our knowledge, no prior study has examined both individual and organizational stress simultaneously using validated instruments in this context. Previous research has been limited by its focus on culturally homogeneous healthcare systems, isolated examination of stress factors, and the lack of effect size quantification to prioritize interventions.^[5,6]^

Moreover, İstanbul presents a unique context. In 2013, the population per EMS station was more than double the national average, and the population per ambulance was 2.3-fold higher than elsewhere in Türkiye.^[7]^ Such extreme service demands highlight the extraordinary stress conditions faced by EMS-HCWs in this city. Importantly, the current study provides valuable pre-pandemic baseline data, offering an essential reference point for future research on stress trajectories under crisis conditions.

## 2. Objectives

The objective of this study was to conduct a comprehensive assessment of both individual and organizational stress levels among EMS-HCWs in İstanbul. Specifically, we aimed to evaluate perceived stress (PSS) and organizational stress (VOS-D) simultaneously, using validated instruments adapted for the Turkish population.^[8–10]^ By identifying workplace, demographic, and behavioral correlates of stress, this study seeks to inform evidence-based, context-appropriate intervention strategies that may also be applicable to other high-density urban EMS systems worldwide.

## 3. Methods

### 3.1. Study design and setting

A cross-sectional analytical study was conducted between September and December 2014 in İstanbul, Türkiye’s most populous city with approximately 15 million inhabitants. The EMS system comprised 194 ambulance stations (122 European side, 72 Asian side) operating 248 emergency rescue ambulances with 1401 personnel (1099 emergency medical technicians and 302 paramedics).^[7]^

#### 3.1.1. Participants

After excluding staff on annual leave and those without current email addresses, 1100 EMS-HCWs were identified as the target population. Convenience sampling was used to recruit all available personnel during the study period, comprising 300 Command and Control Center (CCC) staff and 800 station staff.

Paper-based questionnaires were distributed to CCC staff during shift changes, while electronic surveys were sent to station staff via institutional email. Written informed consent was obtained from CCC staff, and electronic consent was collected online for station staff. All participants received detailed information about study objectives, voluntary participation, anonymity safeguards, and withdrawal rights. No incentives were offered.

**Figure 1.**
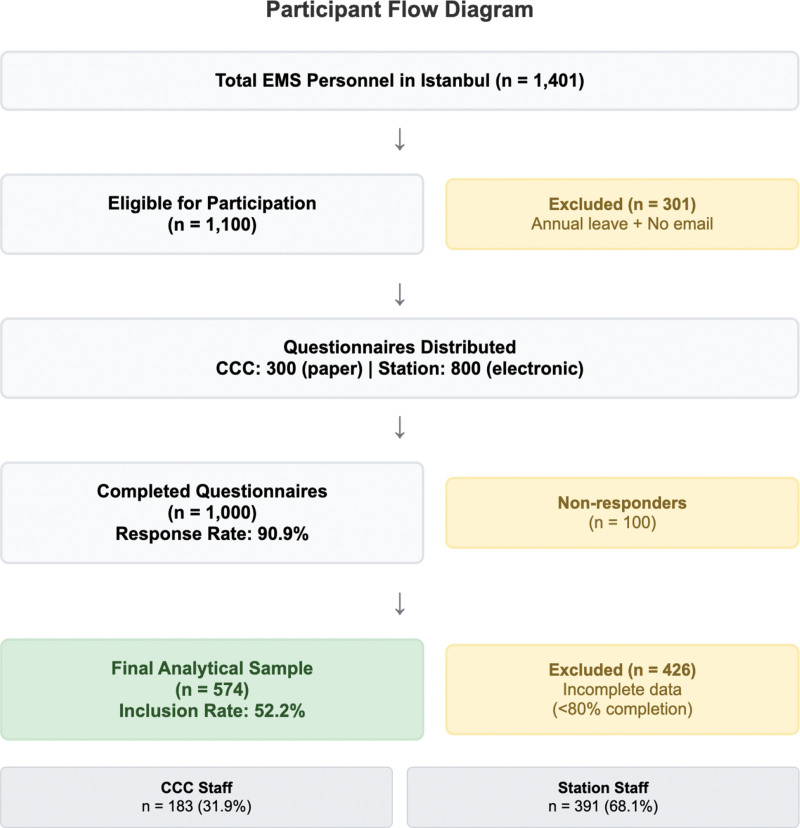
Participant flow diagram of the study population. CCC = Command and Control Center; EMS = emergency medical services.

Of 1000 completed questionnaires received (response rate: 90.9%), responses with <80% item completion were excluded to ensure complete scale scoring, resulting in a final analytical sample of 574 participants (183 CCC, 391 station; inclusion rate: 52.2%; Fig. 1).

#### 3.1.2. Variables

The study questionnaire comprised: 8-item demographic form, 14-item Perceived Stress Scale (PSS-14), 81-item Organizational Stress Questionnaire-Doetinchem (VOS-D), and 7 workplace perception questions.

PSS-14: Measures perceived stress over the past month using a 5-point Likert scale (0 = never to 4 = very often), with total scores ranging 0 to 56. Scores are categorized as low (0–18), moderate (19–37), or high (38–56). The Turkish version demonstrated satisfactory reliability (Cronbach’s α = 0.84).^[8,9]^

VOS-D: An 81-item scale assessing organizational stress across 4 dimensions: stressors, psychological complaints, health problems, and social variables. Items are rated on a 5-point scale (1 = strongly disagree to 5 = strongly agree). The Turkish adaptation showed adequate reliability across subscales (Cronbach’s α: 0.63–0.84).^[10]^

The scoring criteria and stress level classifications for both PSS-14 and VOS-D are presented in Table 1.

**Table 1:** Scoring criteria and stress level classifications for PSS-14 and VOS-D among İstanbul EMS-HCWs (N = 574).

PSS		VOS-D	
Stress level	Score	Stress level	Score conversion to percentiles
Low stress	0–18	Very low stress	Score ≤ 5 percentile
		Low stress	5 percentile < score ≤ 25 percentile
Medium stress	19–38	Medium stress	25 percentile < score < 75 percentile
		High stress	75 percentile ≤ score < 95 percentile
High stress	39–56	Very high stress	95 percentile ≤ score

EMS-HCWs = emergency medical services healthcare workers, PSS = Perceived Stress Scale, VOS-D = Organizational Stress Questionnaire-Doetinchem.

### 3.2. Statistical analysis

Analyses were performed using IBM SPSS version 26.0 (IBM Corp., Armonk). Categorical variables are presented as frequencies and percentages. Normality was assessed using the Shapiro–Wilk test, with normally distributed data presented as mean ± SD and non-normally distributed data as median (min–max). Between-group comparisons used the Mann–Whitney *U* test (2 groups) and Kruskal–Wallis *H* test (multiple groups). Associations were examined using Spearman’s correlation coefficient. Multiple linear regression determined VOS-D subdomain contributions to PSS total scores. Statistical significance was set at *P* < .05.

### 3.3. Ethical considerations

The study was conducted with official permission obtained from the İstanbul Provincial Health Directorate in 2014, prior to data collection. In addition, approval for secondary analysis and publication was granted by the Ethics Committee of İstanbul Training and Research Hospital (Date: July 19, 2024; Decision No. 35).

## 4. Results

The final analytical sample comprised 574 EMS-HCWs with a mean age of 30.98 ± 6.51 years (males: 33.11 ± 7.19; females: 29.21 ± 5.27; *P* < .001). The mean duration of employment in the İstanbul ambulance service was 4.86 ± 4.37 years (males: 5.43 ± 5.10; females: 4.40 ± 3.59; *P* < .05), while the mean total working experience was 8.31 ± 5.64 years (males: 8.75 ± 6.33; females: 7.94 ± 4.97; *P* > .05). Of the EMS-HCWs, 314 (54.7%) were female, 307 (53.5%) were married, 257 (44.8%) were high school graduates, 54 (9.4%) reported alcohol consumption, 266 (46.3%) were smokers, 255 (44.4%) worked in CCC, 319 (55.6%) worked in station units, and 380 (66.2%) were emergency medical technicians.

Overall, 451 participants (78.6%) reported moderate levels of perceived stress. By sex, 258 (82.2%) female EMS-HCWs and 193 (74.2%) male EMS-HCWs were in the moderate stress category.

### 4.1. Stress level distributions by VOS-D components

The distribution of stress levels across VOS-D domains demonstrated varied patterns. Regarding stressors, most participants fell into the moderate category (78.8%), whereas 17.1% were classified as low, 3.0% as high, and only 0.9% and 0.2% as very low and very high, respectively. A similar pattern was observed for psychological complaints, with 78.0% reporting moderate levels, 18.7% low, 2.7% high, and <1% very low or very high levels. In contrast, health problems were predominantly rated as low (62.3%), followed by very low (19.7%), and moderate (17.8%), with virtually no participants in the high category. Regarding social variables, most participants (81.7%) reported moderate stress, 10.6% reported low stress, 4.5% reported high stress, and a small proportion reported very low or very high stress (3.0% and 0.2%, respectively; Table 2).

**Table 2:** Bivariate comparison of VOS-D subfactor scores across PSS levels among EMS-HCWs (N = 574, Kruskal–Wallis analysis).

VOS-D component	PSS low (n = 86)	PSS moderate (n = 451)	PSS high (n = 37)	*H*	*P*
	Mean ± SD	Mean ± SD	Mean ± SD		
Stressors	89.19 ± 10.50	100.13 ± 11.50	110.65 ± 13.15	82.590	<.001***
Psychological complaints	37.78 ± 6.40	47.39 ± 8.49	60.22 ± 9.27	138.267	<.001***
Health problems	23.40 ± 6.82	30.24 ± 9.00	41.27 ± 13.20	97.438	<.001***
Social variables	18.84 ± 5.06	21.92 ± 4.49	24.43 ± 4.22	42.201	<.001***

EMS-HCWs = emergency medical services healthcare workers, H = Kruskal–Wallis *H* test, PSS = Perceived Stress Scale, VOS-D = Organizational Stress Questionnaire-Doetinchem.

*** *P* < .001.

### 4.2. Relationship between PSS and VOS-D components

Significant correlations were observed between the PSS scores and all VOS-D domains, including stressors, psychological complaints, health problems, and social variables (all *P* < .001). Participants with low PSS scores had lower mean values across all domains: stressors (89.19 ± 10.50), psychological complaints (37.78 ± 6.40), health problems (23.40 ± 6.82), and social variables (18.84 ± 5.06). In contrast, those with high PSS scores demonstrated markedly higher means: stressors (110.65 ± 13.15), psychological complaints (60.22 ± 9.27), health problems (41.27 ± 13.20), and social variables (24.43 ± 4.22; Table 2).

### 4.3. Gender differences

Female EMS-HCWs demonstrated significantly higher stress levels across multiple domains than their male counterparts (Table 3). The median total PSS score was higher among females (33 [22–50]) than males (31 [5–46]; *P* < .001). Similarly, females scored significantly higher across all VOS-D components, including stressors (101.39 ± 11.62 vs 96.49 ± 12.93), psychological complaints (49.22 ± 8.80 vs 43.82 ± 9.72), health problems (32.90 ± 10.59 vs 26.33 ± 7.32), and social variables (22.78 ± 4.22 vs 20.21 ± 4.97; all *P* < .001).

**Table 3:** Comparison of PSS and VOS-D scores according to demographic characteristics of EMS-HCWs.

	PSS			VOS-D			
	SE	SP	PSS TOTAL	STR	PSC	HP	SV
Gender (mean ± SD)							
Male	15.95 ± 2.71	15.38 ± 3.48	31.33 ± 5.12	96.49 ± 12.93	43.82 ± 9.72	26.33 ± 7.32	20.21 ± 4.97
Female	15.97 ± 2.36	16.85 ± 2.80	32.82 ± 3.87	101.39 ± 11.62	49.22 ± 8.80	32.90 ± 10.59	22.78 ± 4.22
*U*	40,795.5	**30,168.5**	**32,509**	**30,347.5**	**26,341.5**	**23,982**	**28,236**
*P*	.990	**<**.**001**	**<**.**001**	**<**.**001**	**<**.**001**	**<**.**001**	**<**.**001**
Marital status							
Single	15.75 ± 2.37	16.22 ± 3.10	31.96 ± 4.13	100.24 ± 13.09	47.72 ± 9.90	30.89 ± 10.79	22.27 ± 4.38
Married	16.15 ± 2.64	16.15 ± 3.30	32.31 ± 4.87	98.24 ± 11.84	45.95 ± 9.27	29.09 ± 8.80	21.05 ± 4.98
*U*	**35,167**	60,674.5	37,965	**36,790**	37,117	37,481	**35,131**
*P*	.**003**	.875	.126	.**034**	.051	.077	.**003**
Educational status							
High school	15.84 ± 2.60	15.74 ± 3.47^a^	31.58 ± 5.02^a^	96.59 ± 11.25^a^	44.86 ± 9.24^a^	29.99 ± 10.81^a^^,^^b^	21.29 ± 5.02^a^
Associate degree	15.99 ± 2.45	16.89 ± 2.72^b^	32.89 ± 3.66^b^	100.40 ± 12.99^b^	48.20 ± 9.67^b^	31.25 ± 9.52^b^	22.61 ± 4.05^b^
Licence	16.27 ± 2.69	15.93 ± 3.50^a^	32.20 ± 5.06^a^^,^^b^	101.91 ± 14.00^b^	48.43 ± 10.02^b^	27.89 ± 7.75^a^	20.49 ± 4.90a
Postgraduate	15.86 ± 1.78	16.39 ± 1.99^a^^,^^b^	32.25 ± 2.82^a^^,^^b^	104.00 ± 9.83^b^	48.67 ± 8.59^b^	27.97 ± 7.41^a^^,^^b^	21.81 ± 4.83ab
*H*	1614	**18,427**	**9707**	**24,771**	**18,675**	**9852**	**13,317**
*P*	.656	**<**.**001**	.**021**	**<**.**001**	**<**.**001**	.**020**	.**004**
Alcohol consumption							
Use	15.52 ± 2.47	16.07 ± 2.81	31.59 ± 4.29	101.65 ± 11.13	49.07 ± 9.63	33.81 ± 3.02	23.11 ± 4.17
Not use	16.01 ± 2.52	16.19 ± 3.25	32.20 ± 4.56	98.91 ± 12.57	46.53 ± 9.58	29.52 ± 9.34	21.46 ± 4.78
*U*	**11,677**	13,462.5	11,783	12,243.5	11,948.5	**11,752.5**	**11,430**
*P*	.**040**	.617	.051	.121	.071	.**048**	.**024**
Smoking status							
Use	15.86 ± 2.65	16.24 ± 3.32	32.10 ± 4.91	99.70 ± 11.80	47.31±. 9.62	31.36 ± 10.84	21.71 ± 4.67
Not use	16.05 ± 2.41	16.13 ± 3.11	32.19 ± 4.20	98.71 ± 13.01	46.31 ± 9.58	28.69 ± 8.65	21.54 ± 4.81
*U*	39,926	40,494.5	40,224.5	39,268.5	38,791.5	**35,359.5**	40,301.5
*P*	.597	.812	.708	.392	.273	.**005**	.738
Department of work							
CCC	15.95 ± 2.58	16.49 ± 3.08	32.44 ± 4.10	100.25 ± 13.29	48.69 ± 10.41	31.57 ± 10.45	21.99 ± 5.05
Station	15.98 ± 2.48	15.94 ± 3.29	31.92 ± 4.85	98.30 ± 11.71	45.24 ± 8.62	28.61 ± 9.06	21.32 ± 4.48
*U*	40,656.5	37,140.5	38,516.5	37,611.5	**32,427.5**	**33,040.5**	**36,653.5**
*P*	.993	.072	.273	.121	**<**.**001**	**<**.**001**	.**041**
Task status							
Doctor	16.01 ± 2.21	15.97 ± 3.01^a^	31.99 ± 3.64	103.29 ± 13.29^b^	49.01 ± 9.63^b^	27.20 ± 6.65^a^	20.73 ± 4.94^a^
EMT	15.89 ± 2.64	16.03 ± 3.32^a^	31.92 ± 4.83	97.47 ± 12.47^a^	45.71 ± 9.95^a^	29.51 ± 9.81^a^	21.47 ± 4.90^a^
Paramedic	16.17 ± 2.31	16.84 ± 2.89^b^	33.02 ± 4.00	101.94 ± 10.65^b^	48.75 ± 7.74^b^	33.18 ± 10.82^b^	22.72 ± 3.86^b^
*H*	0.543	**6377**	5066	**27,297**	**21,117**	**17,751**	**8437**
*P*	.762	.**041**	.079	**<**.**001**	**<**.**001**	**<**.**001**	.**015**
Adequate training							
Yes	16.19 ± 2.45	15.94 ± 3.33	32.13 ± 4.70	96.98 ± 11.92	44.83 ± 8.93	28.30 ± 9.42	20.78 ± 4.76
No	15.53 ± 2.66	16.91 ± 2.79	32.44 ± 4.13	104.35 ± 12.42	51.41 ± 9.67	32.85 ± 8.39	23.48 ± 4.26
*U*	**29,248**	**30,100**	34,826.5	**22,897.5**	**21,081.5**	**21,891**	**23,385.5**
*P*	.**002**	.**008**	.962	**<**.**001**	**<**.**001**	**<**.**001**	**<**.**001**
Social support							
Yes	15.75 ± 2.20	14.65 ± 3.49	30.40 ± 4.72	93.35 ± 10.86	40.20 ± 8.73	24.63 ± 7.83	19.10 ± 4.30
No	16.00 ± 2.57	16.46 ± 3.07	32.47 ± 4.43	100.24 ± 12.45	47.98 ± 9.27	30.90 ± 9.83	22.08 ± 4.68
*U*	19,855	**14,399.5**	**15,056.5**	**14,436**	**10,918.5**	**11,682**	**13,408**
*P*	.225	**<**.**001**	**<**.**001**	**<**.**001**	**<**.**001**	**<**.**001**	**<**.**001**
Adequate number of employees							
Yes	16.08 ± 2.06	15.01 ± 3.39	31.09 ± 4.53			24.86 ± 7.70	19.37 ± 4.81
No	15.95 ± 2.65	16.57 ± 3.06	32.52 ± 4.49	101.57 ± 12.44	48.68 ± 9.58	31.56 ± 9.87	22.32 ± 4.53
*U*	29,784.5	**22,030**	**24,452**	**15,679**	**15,139.5**	**15,997**	**19,903**
*P*	.725	**<**.**001**	**<**.**001**	**<**.**001**	**<**.**001**	**<**.**001**	**<**.**001**
The state of job satisfaction							
Yes	16.07 ± 2.45	15.49 ± 3.29	31.56 ± 4.73	93.77 ± 10.31	41.36 ± 6.78	27.21 ± 9.25	20.03 ± 4.65
No	15.89 ± 2.59	16.98 ± 2.94	32.86 ± 4.21	105.28 ± 11.87	52.88 ± 8.58	33.01 ± 9.53	23.37 ± 4.24
*U*	38,320	**30,834.5**	**35,655**	**18,664**	**10,941**	**23,719**	**24,956.5**
*P*	.164	**<**.**001**	**<**.**001**	**<**.**001**	**<**.**001**	**<**.**001**	**<**.**001**
Feeling safe at work							
Yes	16.02 ± 2.26	15.19 ± 3.08	31.22 ± 4.02	93.45 ± 11.33	41.93 ± 8.27	26.32 ± 9.03	19.85 ± 4.70
No	15.97 ± 2.62	16.62 ± 3.17	32.59 ± 4.68	101.70 ± 12.13	48.93 ± 9.37	31.51 ± 9.74	22.37 ± 4.59
*U*	34,612	**25,085.5**	**27,710**	**21,246**	**19,264**	**22,020.5**	**24,016.5**
*P*	.917	**<**.**001**	**<**.**001**	**<**.**001**	**<**.**001**	**<**.**001**	**<**.**001**

The bold values indicate statistically significant differences (*P* < .05) to facilitate readability in this large table.

*H* = Kruskal–Wallis *H* test, HP = health problems, PSC = psychological complaints, PSS TOTAL = total score of Percieved Stress Scale, SE = self-efficacy, SP = stress perception, STR = stressors, SV = social variables, *U* = Mann–Whitney *U* test, VOS-D = Organizational Stress Questionnaire-Doetinchem.

a ,

b The difference between the medians without a common letter is statistically significant (*P* < .05).

### 4.4. Departmental differences

Significant differences were observed between CCC and station staff (Table 3). Command and Control Center workers reported higher scores in psychological complaints (48.69 ± 10.41 vs 45.24 ± 8.62, *P* < .001), health problems (31.57 ± 10.45 vs 28.61 ± 9.06, *P* < .001), and social variables (21.99 ± 5.05 vs 21.32 ± 4.48, *P* = .041). The effect size analysis further indicated medium effects for psychological complaints (*d* = 0.37) and small effects for health problems (*d* = 0.30), whereas the differences in stressor exposure were minimal (*d* = 0.16; Table 4).

**Table 4:** Effect size analysis for bivariate comparisons of stress components by gender and department among EMS-HCWs (N = 574, Cohen’s *d* analysis).

Comparison	Component	Mean ± SD	Cohen’s *d*	Effect size	95% CI	*P*-value
Female vs male	VOS-D stressors	101.39 ± 11.62 vs 96.49 ± 12.93	0.40	Medium	[0.23, 0.57]	<.001
	Psychological complaints	49.22 ± 8.80 vs 43.82 ± 9.72	0.58	Medium	[0.41, 0.75]	<.001
	Health problems	32.90 ± 10.59 vs 26.33 ± 7.32	0.70	Large	[0.53, 0.87]	<.001
	Social variables	22.78 ± 4.22 vs 20.21 ± 4.97	0.55	Medium	[0.38, 0.72]	<.001
	PSS stress perception	16.85 ± 2.80 vs 15.38 ± 3.48	0.47	Medium	[0.30, 0.64]	<.001
	PSS self-efficacy	15.97 ± 2.36 vs 15.95 ± 2.71	0.01	Negligible	[−0.16, 0.18]	.990
	PSS total	32.82 ± 3.87 vs 31.33 ± 5.12	0.34	Small–Medium	[0.17, 0.51]	<.001
CCC vs station	VOS-D stressors	100.25 ± 13.29 vs 98.30 ± 11.71	0.16	Small	[−0.01, 0.33]	.121
	Psychological complaints	48.69 ± 10.41 vs 45.24 ± 8.62	0.37	Small–Medium	[0.20, 0.54]	<.001
	Health problems	31.57 ± 10.45 vs 28.61 ± 9.06	0.30	Small	[0.13, 0.47]	<.001
	Social variables	21.99 ± 5.05 vs 21.32 ± 4.48	0.14	Small	[−0.03, 0.31]	.041

Effect sizes interpreted as: Small (*d* = 0.2), Medium (*d* = 0.5), Large (*d* = 0.8).

CCC = Command and Control Center, EMS-HCWs = emergency medical services healthcare workers, PSS = Perceived Stress Scale, VOS-D = Organizational Stress Questionnaire-Doetinchem.

### 4.5. Workplace and behavioral correlates

Several workplace and behavioral factors were significantly associated with stress levels (Table 3). Emergency medical services healthcare workers who reported receiving adequate training demonstrated lower scores across all VOS-D domains (all *P* < .001). Similarly, the absence of social support, perceived inadequate staffing, dissatisfaction with work, and feeling unsafe in the workplace were associated with significantly higher stress scores across all domains (all *P* < .001). Current smokers had higher scores on the health problems domain (*P* = .005). Alcohol consumption was associated with significantly higher scores for health problems (*P* = .048) and social variables (*P* = .024), whereas differences in PSS total, stressors, and psychological complaints were not statistically significant.

### 4.6. Predictive model for perceived stress

Multiple linear regression analysis revealed that VOS-D components significantly predicted PSS total scores, explaining 23.4% of the variance (*F* = 43.6, *P* < .001, Table 5). Stressors (β = 0.048, *P* = .015), psychological complaints (β = 0.094, *P* < .001), and social variables (β = −0.093, *P* = .039) were significant predictors, whereas health problems were not statistically significant (β = 0.030, *P* = .175).

**Table 5:** Multiple linear regression analysis of organizational stress predictors for perceived stress total scores among EMS healthcare workers (N = 574, *R*^2^ = 0.234).

Predictor	Unstandardized coefficients β	SE	*t*	*P*	95% confidence interval
					Lower–upper
Constant	24.084	1.473	16.351	<.001*	21.191–26.978
Stressors	0.048	0.020	2.447	.015*	0.009–0.087
Psychological complaints	0.094	0.028	3.318	<.001*	0.038–0.150
Health problems	0.030	0.022	1.357	.175	−0.014 to 0.074
Social variables	−0.093	0.045	−2.072	.039*	−0.182 to −0.005

Model: *R*^2^ = 0.234, *F* = 43.6, *P* < .001.

β = beta coefficient, EMS = emergency medical services, SE = standard error.

* *P* < .05.

## 5. Discussion

### 5.1. Summary of key findings

This study revealed a high prevalence of moderate perceived stress (78.6%) and organizational stress among EMS-HCWs in İstanbul. Key factors associated with higher stress levels include being female, working in CCC, lower educational attainment, lack of adequate training, insufficient social support, and negative perceptions of workplace safety and staffing levels. Behavioral factors such as smoking and alcohol use were also linked to elevated stress scores.

### 5.2. Comparison with existing literature

The high prevalence of moderate to high stress levels among EMS-HCWs in this study is consistent with previous research. Petrie et al^[5]^ reported a pooled prevalence of post-traumatic stress disorder (PTSD) of approximately 10% among ambulance personnel globally, while Ntatamala and Adams^[2]^ highlighted barriers to mental health care access for ambulance staff, underscoring the widespread psychological burden in this workforce. Although PTSD and perceived stress represent different constructs, both reflect substantial psychological strain associated with prehospital emergency work. Similarly, Abbaspour et al^[11]^ reported a high prevalence of mental health problems among EMS-HCWs in Iran, reinforcing the conclusion that EMS-HCWs face significant psychological challenges in diverse contexts. Our finding of 78.6% moderate perceived stress indicates a higher overall burden than global estimates and exceeds the rates reported in previous international studies.

The sex differences observed in our study, with female EMS-HCWs reporting significantly higher stress levels, are consistent with the previous findings. Prior research has highlighted that female EMS-HCWs may be more vulnerable to occupational stress, potentially due to the interplay between societal expectations, coping strategies, and workplace dynamics. Visser et al^[12]^ reported higher stress and burnout among female physicians in the Netherlands, whereas Bennett et al^[13]^ documented higher rates of mental health problems among female ambulance workers in the United Kingdom. Similarly, Rybojad et al^[14]^ identified the female sex as a risk factor for PTSD among Polish paramedics. Taken together, these findings reinforce the conclusion that sex-related factors play an important role in shaping vulnerability to stress in emergency medical settings.

The elevated stress levels observed among CCC staff in our study are consistent with the findings of Okamoto et al,^[15]^ who reported higher rates of burnout and psychological complaints among communication center operators than among field-based firefighters in Japan. Although CCC staff members are less exposed to physical hazards, their substantial cognitive load, continuous decision-making demands, and frequent exposure to secondary trauma likely contribute to their increased vulnerability to stress.

The prevalence of stress was significantly lower among employees who reported feeling safe at work than those who did not. Similarly, Dadashzadeh et al^[16]^ identified trauma exposure, resuscitation, and driving under critical conditions as major stressors for EMS-HCWs. Although their study did not directly assess perceived workplace safety, these operational challenges may undermine feelings of safety, thereby contributing to elevated stress levels.

Workplace factors emerged as critical determinants of perceived stress, consistent with evidence that working conditions exert a greater influence on stress levels than individual characteristics.^[17,18]^ Importantly, sustained organizational stressors such as high workload and insufficient resources not only exacerbate stress, but also reduce organizational commitment among healthcare workers.^[19]^ Inadequate staffing and insufficient supervisor support have been identified as strong predictors of stress among EMS and emergency department staff, underscoring the central role of the organizational context in shaping occupational well-being. Beyond these structural influences, dynamic psychosocial risk and protective factors such as resilience, social support, and coping mechanisms also play a decisive role in determining stress outcomes among EMS-HCWs.^[20]^

Our study also identified lower educational attainment as a factor that is associated with higher stress levels. Previous research suggests that individuals with lower formal education may have limited access to adaptive coping strategies and fewer opportunities for professional development, which can amplify their vulnerability to occupational stress. Similarly, studies among healthcare workers in different contexts have reported an inverse relationship between educational level and perceived stress, highlighting the potential protective role of advanced training and continuing education opportunities.^[17,18]^

Interestingly, while most domains of VOS-D demonstrated moderate stress levels, the “health problems” domain was predominantly rated as low (62.3%). This divergence may reflect the relatively young average age of the EMS workforce in our study (mean 30.9 years), where physical health complaints are less prevalent than psychological and organizational stressors. Alternatively, lower reporting of health-related problems may indicate under-recognition or under-reporting due to the stigma surrounding health complaints among EMS-HCWs. Similar findings have been reported in other occupational health studies, in which psychological stress was more readily acknowledged than physical symptoms.^[17]^

Regression analyses further demonstrated that stressors, psychological complaints, and social variables were significant predictors of perceived stress, while health problems were not. This pattern underscores the dominant role of the organizational and psychosocial domains in shaping perceived stress among EMS personnel. Bardhan and Byrd^[18]^ similarly emphasized that workplace dynamics and interpersonal stressors outweigh somatic complaints in predicting stress outcomes. Moreover, Hruska and Barduhn^[19]^ highlighted that psychosocial risk and protective factors, including resilience and coping mechanisms, are key determinants of mental health in EMS personnel, supporting the importance of the organizational and social dimensions identified in our study. Importantly, both references reflect post-pandemic evidence, demonstrating that these psychosocial and organizational stress mechanisms have remained prominent among EMS-HCWs beyond the COVID-19 period.^[18,19]^

The prevalence of moderate stress (78.6%) observed in our study (Table 2) was notably higher than that reported in comparable populations.^[5,6]^ This suggests that urban density and workload pressures in transcontinental megacities such as İstanbul may contribute to distinctive stress profiles. The finding that İstanbul’s population per ambulance (52,253) is more than twice the national average (2.3-fold higher) provides important contextual evidence for interpreting elevated stress levels.^[7]^

Behavioral factors such as smoking and alcohol use, which were associated with higher stress in our study, align with previous findings that EMS-HCWs often adopt maladaptive coping strategies, including substance use, to manage work-related stress.^[2,20]^

## 6. Strengths and limitations

### 6.1. Strengths

This study had several methodological strengths that enhanced its contribution to the literature. The large sample size (n = 574) provided adequate statistical power for robust analysis of stress patterns among EMS-HCWs. The use of validated instruments (PSS-14 and VOS-D), previously adapted for Turkish populations, ensured cultural appropriateness and measurement reliability. A key strength is the simultaneous assessment of both individual perceived and organizational stress factors, a combination rarely achieved in EMS research. The comprehensive recruitment strategy achieved a high response rate (90.9%), thereby minimizing potential selection bias. Additionally, this study provides valuable pre-pandemic baseline data from 2014, which is essential for understanding how extraordinary circumstances, such as the COVID-19 pandemic, amplify existing stress patterns in EMS-HCWs.

### 6.2. Limitations

Several limitations should be considered when interpreting our findings. The time frame of data collection (2014) may limit direct applicability to current EMS contexts, particularly following extraordinary stressors such as the COVID-19 pandemic. Recent studies have demonstrated marked declines in morale, increased stress, and deteriorating mental health among EMS-HCWs during the pandemic, highlighting the substantial amplification of occupational stress under crisis conditions.^[21]^ Qualitative research from Iran has shown that EMS-HCWs adopt diverse coping strategies to manage pandemic-related stressors, underscoring the need for contextually sensitive support mechanisms.^[22]^ Furthermore, Bardhan and Byrd reported that organizational and psychosocial stressors remained prominent among EMS-HCWs in the post-pandemic era, reinforcing the relevance of our findings and suggesting that preexisting stress patterns may have intensified over time.^[18]^

The strict data quality criteria resulted in an inclusion rate of 52.2% (574/1100) as responses with missing data were excluded to ensure complete scale scoring. While this approach maintains methodological rigor, it may introduce potential bias if the excluded participants differ systematically from those included.

Regarding generalizability, while the sample is comprehensive for İstanbul, the findings may be most applicable to other metropolitan centers with similarly high case loads per healthcare worker and comparable organizational pressures, both within Türkiye and in EMS systems in other global urban contexts where similar cultural and operational dynamics exist.

## 7. Implications for practice and future research

These findings highlight the urgent need for multifaceted interventions to reduce occupational stress in EMS-HCWs. Organizational strategies should prioritize enhancing workplace safety, implementing routine mental health screening, offering resilience and coping skills training, improving staffing ratios, and fostering supportive peer and supervisory relationships.

Future research should build on this study by using several complementary approaches. Longitudinal assessments can track stress trajectories before and after major healthcare crises, providing insights into how extraordinary circumstances amplify the existing stress patterns. Intervention studies should design and test culturally appropriate programs, particularly targeting high-risk groups such as female EMS-HCWs and CCC staff, who demonstrated elevated stress levels in this study. Multi-center replication studies in other high-density urban EMS settings would help examine generalizability and explore the specific organizational and cultural mechanisms underlying gender- and department-related disparities.

Economic evaluations of workplace interventions targeting modifiable risk factors would provide valuable evidence for policy decision-making and resource allocation. Complementary qualitative approaches could further deepen our understanding of EMS-HCWs’ lived experiences of stress across diverse sociocultural contexts, offering insight into contextually sensitive intervention strategies.

Taken together, these research directions can inform evidence-based policy and practice initiatives aimed at safeguarding the psychological well-being of EMS-HCWs in high-demand urban environments, ultimately supporting workforce sustainability and the delivery of high-quality emergency care.

## 8. Conclusion

This study demonstrated that EMS-HCWs in İstanbul experience significant levels of work-related stress, particularly among female EMS-HCWs, CCC staff, and those reporting inadequate training or limited social support. These findings call for immediate action by EMS administrators and policymakers to implement evidence-based, context-specific interventions that strengthen workplace safety, optimize staffing ratios, enhance resilience and coping skills, and improve peer and supervisory support. Addressing these factors is essential for protecting the well-being of EMS-HCWs and ensuring workforce sustainability. Ultimately, such measures will safeguard both workforce resilience and delivery of high-quality emergency care in high-demand urban environments.

## Acknowledgments

The authors thank all emergency medical service personnel who participated in this study for their valuable contributions. The authors acknowledge the support of the İstanbul Provincial Health Directorate and affiliated institutions, which were essential for the completion of this study.

## Author contributions

**Conceptualization:** Derya Abuşka, Yilmaz Aydin, Doğaç Niyazi Özüçelik.

**Data curation:** Derya Abuşka, Hasan Yasin Soylu.

**Formal analysis:** Derya Abuşka.

**Methodology:** Derya Abuşka, Yilmaz Aydin, Verda Tunaligil, Doğaç Niyazi Özüçelik.

**Project administration:** Verda Tunaligil.

**Supervision:** Yilmaz Aydin, Verda Tunaligil, Doğaç Niyazi Özüçelik.

**Validation:** Hasan Yasin Soylu.

**Writing – original draft:** Derya Abuşka, Yilmaz Aydin.

**Writing – review & editing:** Derya Abuşka, Yilmaz Aydin, Hasan Yasin Soylu, Verda Tunaligil, Doğaç Niyazi Özüçelik.
